# Urinary Obstruction of Transplanted Kidney Caused by Uterine Adenomyosis and 2-Year Posthysterectomy Psoas Abscess in Conjunction with Transplanted Kidney

**DOI:** 10.1155/2016/7142537

**Published:** 2016-12-20

**Authors:** Yuta Takezawa, Yoshifumi Kadono, Takahiro Nohara, Atsushi Mizokami

**Affiliations:** Department of Urology, Kanazawa University Hospital, 13-1 Takara-machi, Kanazawa, Japan

## Abstract

Urinary obstruction of the transplanted kidney caused by uterine leiomyoma is an extremely rare condition. To the best of our knowledge, there are only two reports in English literature. Psoas abscess secondary to renal graft pyelonephritis is also uncommon. We present this unusual case and its treatment course. A 43-year-old female presented with renal dysfunction. She was started on peritoneal dialysis from the age of 26 years and received kidney transplantation from her mother (living donor) at the age of 27 years. Computed tomography (CT) revealed right hydronephrosis and a large uterine mass compressing the distal ureter of the transplanted kidney. After a simple total hysterectomy, her renal function improved. Two years following the hysterectomy, she experienced painful urination, fever, right abdominal pain, and right lower limb pain. CT and T2-weighed magnetic resonance imaging of her pelvis demonstrated right psoas abscess in conjunction with transplanted kidney. She was treated with broad-spectrum antibiotics alone, which resulted in a good response. Urinary obstruction of the transplanted kidney caused by uterine leiomyoma is an extremely rare condition. Psoas abscess secondary to transplanted kidney pyelonephritis is also rare. We should keep these rare diseases in mind when treating such cases.

## 1. Introduction

Ureteral obstruction and urinary leak represent the most frequent urological complications after renal transplantation [1]. The incidence of transplant ureteric obstruction is 2%–5% among all transplants, and most of these occur within the first 4 months after surgery [1, 2]. The most common cause of urinary obstruction is ureteric stenosis because of the surgical technique used; other causes include edema, ischemia, necrosis of the ureter, intraluminal blood clot, lymphocele, and hematoma [2]. Urinary obstruction of the transplanted kidney caused by uterine leiomyoma is an extremely rare condition. To the best of our knowledge, there are only two reports in the English literature [2, 3]. There is one report of an ovarian tumor causing urinary obstruction [4]. Furthermore, the present case also suffered from psoas abscess secondary to graft pyelonephritis 2 years after the operation. Psoas abscess is also a rare condition, but it is now diagnosed and reported more frequently because of the increased use of computed tomography (CT) scans [5]. However, psoas abscess secondary to renal graft pyelonephritis is extremely uncommon. We present this unusual case and its treatment course.

## 2. Case Report

**Figure 1:**
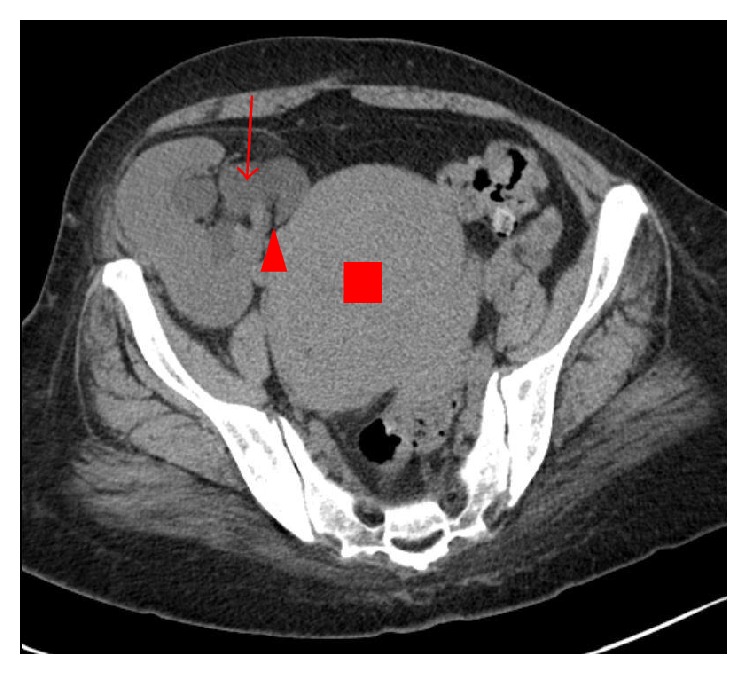
Plain computed tomography. A large uterine tumor (red square) compressed the distal ureter of the transplanted kidney (red arrowhead), causing hydronephrosis (red arrow).

A 43-year-old female who received kidney transplantation from a living donor presented with renal dysfunction. Her history revealed purpura nephritis, which was diagnosed at the age of 2 years and culminated in end-stage renal disease at the age of 26 years. She was started on peritoneal dialysis from the age of 26 years, and she received kidney transplantation from her mother (living donor) at the age of 27 years. Her renal dysfunction steadily progressed from the age of 42 years, and she was referred to our department to investigate the cause of the renal dysfunction. She was given cyclosporine, azathioprine, and prednisolone. Physical examination was normal. Blood pressure was 152/95 mmHg, heart rate was 90 beats/min, body temperature was 36.7°C, and Body Mass Index (BMI) was 29.8. On laboratory examination, she had anemia (Hb, 6.6 g/dl), her serum creatinine (Cr) level was 1.88 mg/dl, and her urine was clear. CT revealed a large uterine mass compressing the distal ureter of the transplanted kidney and causing hydronephrosis (Figure 1).

First, a 5 Fr ureteral stent was retrogradely placed in the transplanted ureter. However, a percutaneous nephrostomy was needed because the serum Cr levels and hydronephrosis of the transplanted kidney did not improve. Serum Cr and hydronephrosis immediately improved after the nephrostomy, but it was necessary to perform a simple hysterectomy to remove the nephrostomy tube. The hysterectomy was performed a month later, and the pathological diagnosis was uterine adenomyosis.

**Figure 2:**
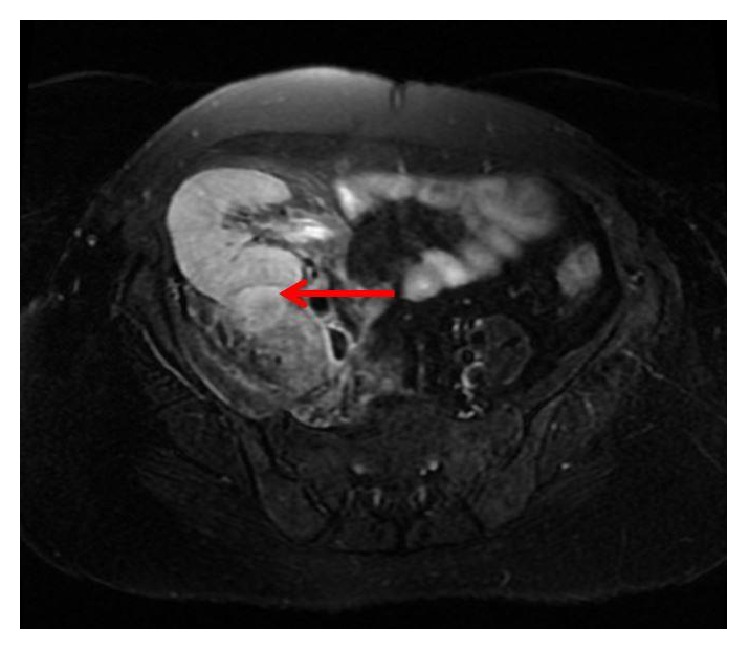
Contrasted T2-weighted magnetic resonance imaging. Right psoas abscess was in conjunction with the transplanted kidney. Ring-enhancement lesion was exhibited on magnetic resonance imaging (red arrow).

**Figure 3:**
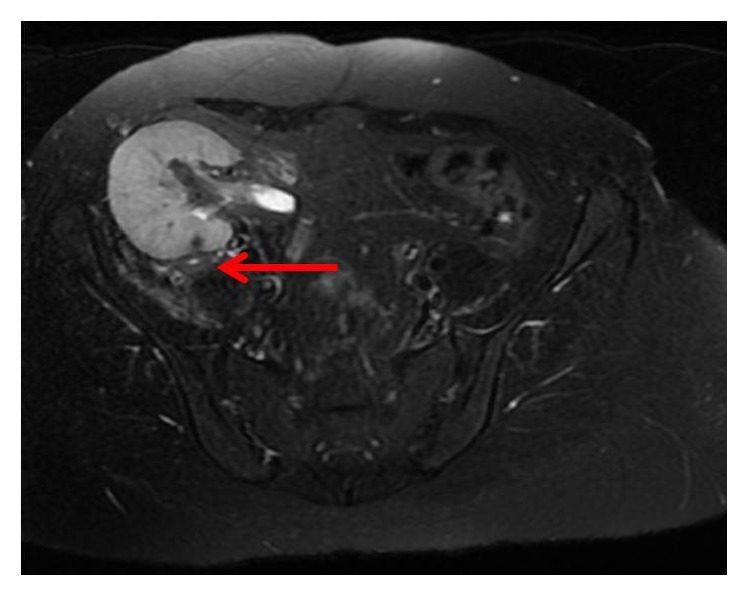
Contrast T2-weighted magnetic resonance imaging. After the patient was given antibiotics, right psoas abscess ameliorated within 2 months (red arrow).

Two years after the hysterectomy, she experienced painful urination, fever, right abdominal pain, and right lower limb pain. Physical examination revealed right abdominal tenderness on the transplanted kidney. Her blood pressure was 120/85 mmHg, heart rate was 110 beats/min, and body temperature was 38.5°C. Her white blood cell count was 1.32 × 10^4^/*μ*l, C-reactive protein level was 14.4 mg/dl, and serum Cr level was 2.73 mg/dl. Urinalysis revealed pyuria. T2-weighed magnetic resonance imaging (MRI) of her pelvis demonstrated right psoas abscess in conjunction with the transplanted kidney (Figure 2). Immediately, broad-spectrum antibiotics were administered. *β*-Streptococcus was isolated from the urine culture. The blood bacterial culture was negative. Her fever and abdominal pain were quickly alleviated, and T2-weighed MRI after 2 months revealed abscess improvement (Figure 3). Immunosuppressive agent was not changed during the entire treatment course. No relapses have occurred after the completion of treatment.

## 3. Discussion

The most common cause of urinary obstruction is ureteric stenosis because of the surgical technique used; other causes include edema, ischemia, necrosis of the ureter, intraluminal blood clot, lymphocele, and hematoma [2]. Two reports of the extremely rare urinary obstruction of the transplanted kidney caused by uterine leiomyoma were found in the English literature [2, 3]. However, a clinician must consider this rare condition, which could be fatal for patients with a transplanted kidney, when making an early treatment plan. In the present case, the patient underwent ureteral stent placement, which was ineffective because of the severe ureteral stenosis, and a percutaneous nephrostomy was needed. A simple hysterectomy was then performed. Previously, two reported cases of urinary obstruction caused by uterine tumor also involved a simple total hysterectomy [2, 3]. We should keep this option in mind to improve and maintain the transplanted kidney function. If the diagnosis was possible before transplantation, the patient may need to have performed preoperative enucleation or hysterectomy.

Psoas abscess secondary to renal graft pyelonephritis is also uncommon [5–7]. Intravenous drug use, human immunodeficiency virus, and Acquired Immune Deficiency Syndrome (AIDS) are emerging as common risk factors for the development of primary psoas abscesses [8]. A previous report stated that Crohn's disease is the most common cause of secondary psoas abscess in the western world [9]. In the present case, the patient was diagnosed with a psoas abscess secondary to the pyelonephritis of the transplanted kidney. The patient underwent a simple total hysterectomy, but the postoperative status was not related to the cause of psoas abscess because it occurred 2 years after the hysterectomy. The urine culture revealed *β*-streptococcus, while the blood bacterial culture was negative.* S. aureus* is implicated in most cases with primary psoas abscess. Other causative organisms include* Streptococcus* sp.,* Escherichia coli*,* Mycobacterium tuberculosis*,* Mycoplasma pneumonia*,* Proteus*,* Pasteurella*, and* Bacteroides* spp. [5, 6]. In our case, the pathogens of the psoas abscess were not identified; however, it was considered to be a secondary event leading to pyelonephritis by reasons of the positive urine bacterial culture, the negative blood bacterial culture, and the adjacent position of infected transplanted kidney and psoas abscess. Yacoub et al. reported that psoas abscess should initially be nonsurgically treated with antibiotics, with or without percutaneous drainage. The administration of antibiotics alone for abscesses smaller than 3 cm in the largest diameter and percutaneous drainage for those larger than 3 cm could be recommended. Surgical drainage is a rare treatment option [5]. In our case, the diameter of the abscess was smaller than 3 cm; therefore, the administration of broad-spectrum antibiotics without drainage was selected as the first-line treatment and was greatly effective. We need to carefully consider the treatment plan, taking into account the patient's systematic condition and abscess size.

## 4. Conclusions

Urinary obstruction of the transplanted kidney caused by uterine leiomyoma is an extremely rare condition. Psoas abscess secondary to transplanted kidney pyelonephritis is also rare. We should keep these rare diseases in mind when treating such cases.

## Abbreviations

AIDS: Acquired Immune Deficiency Syndrome
BMI: Body Mass Index
Cr: Creatinine
CT: Computed tomography
MRI: Magnetic resonance imaging.
